# Stage-associated overexpression of the ubiquitin-like protein, ISG15, in bladder cancer

**DOI:** 10.1038/sj.bjc.6603099

**Published:** 2006-04-25

**Authors:** J B Andersen, M Aaboe, E C Borden, O G Goloubeva, B A Hassel, T F Ørntoft

**Affiliations:** 1 University of Maryland, Marlene and Stewart Greenebaum Cancer Center, 655 West Baltimore Street, 9th floor BRB, Baltimore, MD 21201, USA; 2 Molecular Diagnostic Laboratory, Department of Clinical Biochemistry, Aarhus University Hospital, Aarhus 8200 N, Denmark; 3 The Cleveland Clinic Foundation, Cleveland, OH 44195, USA; 4 Department of Microbiology and Immunology, University of Maryland, 655 West Baltimore Street, 9th floor BRB, Baltimore, MD 21201, USA

**Keywords:** bladder cancer, interferon, ISG15, ubiquitin-like protein, inflammation

## Abstract

Bladder cancer is among the most prevalent malignancies, and is characterised by frequent tumour recurrences and localised inflammation, which may promote tissue invasion and metastasis. Microarray analysis was used to compare gene expression in normal bladder urothelium with that in tumours at different stages of progression. The innate immune response gene, interferon-stimulated gene 15 kDa (ISG15, *GIP2*), was highly expressed at all stages of bladder cancer as compared to normal urothelium. Western blotting revealed a tumour-associated expression of ISG15 protein. ISG15 exhibited a stage-associated expression, with significantly (*P*<0.05) higher levels of ISG15 protein in muscle-invasive T2–T4 tumours, compared with normal urothelium. Although ISG15 is involved in the primary immune response, ISG15 expression did not correlate with bladder inflammation. However, immunohistochemical staining revealed expression of ISG15 protein in both cancer cells and stromal immune cells. Interestingly, a significant fraction of ISG15 protein was localised to the nuclei of tumour cells, whereas no nuclear ISG15 staining was observed in ISG15-positive stromal cells. Taken together, our findings identify ISG15 as a novel component of bladder cancer-associated gene expression.

Bladder cancer in the US is ranked the fourth most prevalent cancer type among men (6%) and tenth among females (2%), and similar figures are found in the rest of the industrialised world (Jemal *et al*, 2003). The average survival rate 1 year after diagnosis is 81%, 66% after 5 years, and 57% after 10 years. If bladder cancer is diagnosed at an early stage in development when the tumour is confined to the bladder, the 5-yearsurvival rate is 94%; however, if the malignancy is not detected until it has spread regionally beyond the bladder or as distal metastases, the survival rate drops to 48 and 6%, respectively (The American Cancer Society, 2003). Recurrence is a particular risk in bladder cancer as tumours are frequently accompanied by precancerous genetic alterations in the surrounding, morphologically normal urothelium (Rao *et al*, 1993; Hartmann *et al*, 1999; Czerniak *et al*, 2000). Bladder tumours classified as benign mucosal (Ta) and submucosal invasive (T1) comprise 80% of initial patient diagnoses; the remaining 20% of patients present T2–T4 tumours that eventually develop distal metastases (Dyrskjot, 2003). Invasion of bladder tumour cells into the submucosa is accompanied by a marked host immune response; the resultant localised cell killing is thought to further promote tumour spread. The host immune system plays an important role in tumour detection and eradication (Dunn *et al*, 2004). Similar to the response to foreign agents, phagocytic and antigen presenting cells of the innate immune system function to detect endogenous malignantly transformed cells. An innate immune response is initiated through the direct contact of immune cells with malignant cells, or via activation of Toll-like receptors by tumour-derived molecules, and results in the transcriptional induction of immune response genes. Consistent with this scenario, recent studies have determined that molecular components of the innate immune response are critical for host antitumour activity (Shankaran *et al*, 2001; Akazawa *et al*, 2004; Zheng *et al*, 2004), and that exogenous activators of innate immunity are efficacious antitumour agents in an adjuvant therapy setting (Krieg, 2004).

Interferon-stimulated gene 15 kDa (ISG15) is a interferon-inducible ubiquitin-like protein and its expression is highly induced upon viral or bacterial infection (Loeb and Haas, 1992; Kim and Zhang, 2003). Type I interferons (IFNs) induce several hundred IFN-stimulated genes, including ISG15, through the Janus kinase/signal transducer and activator of transcription (Jak/STAT) signalling pathway. Interferon alpha and beta are the strongest inducers of the ISG15 gene (Der *et al*, 1998). The ISG15 protein is comprised of tandem ubiquitin homology domains (Haas *et al*, 1987; Potter *et al*, 1999) with distinct intracellular and extracellular activities. Within cells, ISG15 is covalently conjugated to cellular proteins in an enzymatic pathway similar to that used by ubiquitin. In contrast to ubiquitin, post-translational modification by ISG15 does not result in the proteasome-dependent degradation of ISG15 conjugates. Multiple target proteins of ISG15 have recently been identified; however, the biological consequences of modification by ISG15 remain to be determined (Hamerman *et al*, 2002; Malakhov *et al*, 2003; Giannakopoulos *et al*, 2005; Zhao *et al*, 2005). Extracellular ISG15 is proposed to have a cytokine-like function by activating CD3+ T cells (D'Cunha *et al*, 1996a, 1996b). Most recently, ISG15 was identified in a screen of red blood cell lysates for neutrophil chemotactic factors, suggesting a novel role for extracellular ISG15 (Owhashi *et al*, 2003).

In an effort to identify changes in gene expression associated with bladder cancer, microarray analysis was used to compare the mRNA profile in normal bladder urothelium with that in tumours from different stages of progression. We focused on immune response genes to investigate their relationship to bladder cancer-associated inflammation. The ISG15 gene was significantly upregulated in bladder tumours as compared to normal tissues, and ISG15 protein increased with advancing stage of the bladder cancer. Importantly, ISG15 expression did not correlate with a generalised inflammatory response, suggesting that it is specifically associated with bladder tumours. Indeed, immunohistochemical staining showed ISG15 expression in both cancer cells and stromal immune cells. Our findings indicate that elevated expression of ISG15 is a novel feature of bladder cancer.

## MATERIALS AND METHODS

### Tissue material

In total, 138 bladder tumours and 27 normal urothelium biopsies from healthy individuals were obtained by surgery and frozen immediately in a preserving solution of guanidinium thiocyanate and stored at −80°C. Informed consent was obtained in all cases, and protocols were approved by the local scientific ethical committee (Internal Review Board).

### DNA microarray analysis

Preparation of labelled cRNA, microarray hybridisation, washing, and scanning was performed according to the manufacturer's instructions as described previously (Dyrskjot *et al*, 2003). Two types of expression microarrays were used in the study, affymetrix HG-U133A microarrays and customised affymetrix microarrays (EOS Hu03) designed by EOS Biotechnology Inc. (now PDL BioPharma, Fremont, CA, USA). A single EOS Hu03 microarray contains >59 000 probesets, which represent approximately 45 000 genes/ESTs, as well as 6 900 *ab initio* predicted genes not represented in human genome sequences at the time of chip design (Eaves *et al*, 2002).

### Immunohistochemistry

For immunohistochemical staining, either 5-*μ*m paraffin-embedded tissue sections or bladder tissue microarrays were used. Bladder tissue microarrays contained normal urothelium (*N*=10), Ta tumour (*N*=20), and T2–T4 tumour (*N*=20). The tissue sections were deparaffinised, rehydrated, boiled in a microwave oven for 5 min, allowed to cool at room temperature for 20 min, and then incubated in methanol/H_2_O_2_ (100 ml methanol+1.5 ml (w v^−1^) H_2_O_2_) for 10 min to block endogenous peroxidase. The tissue sections were then incubated in TEG buffer (Tris-EGTA, pH 9.0) boiled for 10 min in a microwave, allowed to cool at room temperature for 20 min, and incubated with Pronase DAKO S2013 (DakoCytomation A/S, Glostrup, Denmark) for 20 min at room temperature. Then, tissue sections were incubated with rabbit polyclonal anti-ISG15 antibody (30 *μ*g ml^−1^) and visualised using the Envision™ visualisation system. As chromogen, we used DAB+ from DAKO (K3868; DakoCytomation A/S, Glostrup, Denmark), and counterstained with Mayer's haematoxylin and mounted in Aquatex® (1.08562; Merck, Darmstadt, Germany). The evaluation of the tissue microarrays was performed independently by two individuals (MAJ, LDA). The ISG15 expression was scored as being present or not. Nuclear staining of urothelial and cancer cells was scored positive when more than 50% of the nuclei were ISG15 positive. Unspecific tissue border staining was excluded from the analysis.

### Western blot analysis

Tissue lysates were prepared using radioimmunoprecipitation assay buffer (150 mM sodium chloride, 1.0% Nonidet P-40, 0.5% sodium deoxycholate, 0.1% sodium dodecyl sulphate (SDS), 50 mM Tris, pH 8.0; Upstate, Charlottesville, VA, USA). The protein concentration in the lysates were determined by the Bradford microassay (Bio-Rad, Hercules, CA, USA), and 10-100 *μ*g of lysate protein was separated on 12% SDS–polyacrylamide gel electrophoreses gel for analysis of ISG15 protein and ISG15 conjugates. Proteins were electrotransferred to Immobilon-P membrane (Millipore, Billerica, CA, USA). The membranes were blocked in 5% nonfat milk TBST buffer (10 mM Tris, pH 8.0, 150 mM NaCl, 0.1% v v^−1^ Tween 20) for 1 h at room temperature and then sequentially reacted to the primary antibody (mouse monoclonal anti-ISG15 antibody) for 1 h in blocking buffer and the horseradish peroxidase-conjugated secondary antibody (1 : 10 000 dilution; Sigma-Aldrich, St Louis, MO, USA). The immunoreactive complex was visualised by the Pierce SuperSignal chemiluminescent substrate (Pierce, Rockford, IL, USA) and exposure to X-Omat AR film (Kodak, Rochester, MN, USA).

### Bioinformatics

Microarray data for ISG15 expression profiling was either normalised using the RMA procedure (Bolstad *et al*, 2003) (HG-U133A) or by procedures previously described in Dyrskjot *et al* (2005) (Eos Hu03 microarray). Two independent samples sets were analysed by either affymetrix HG-U133A arrays (*N*=54; nine normal urothelium and 45 Ta tumours) or custom affymetrix arrays (*N*=111 Eos Hu03 microarrays; 18 normal urothelium, 28 Ta tumour, 20 T1 tumours, and 45 T2–T4 tumours). Pairwise comparisons of levels of ISG15 protein expression between normal and tumour samples were performed using Student's *t*-test. Pearson's correlation coefficients (*ρ*) were calculated in Microsoft Excel and *P*=0.001 levels of significance were calculated for both data sets using the formula: *R*=(*U*−1)/(*U*+1), where 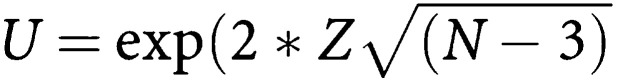, Z(0.001)=3.09, *N*=54, or *N*=111. Only genes with median expression of more than 50 were included in the analysis.

## RESULTS

### ISG15 gene transcript is increased in bladder cancer

**Figure 1:**
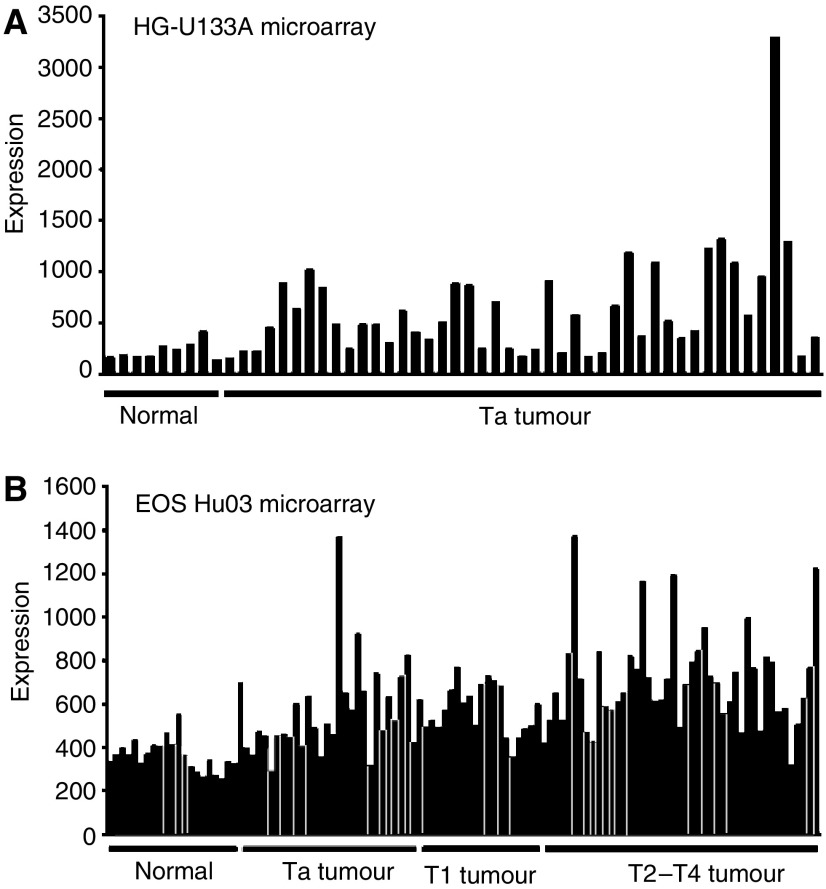
Microarray analysis of ISG15 gene expression in normal urothelium and bladder tumour. (**A**) ISG15 gene expression in normal urothelium (*n*=9) and in tumours from patients with Ta grade bladder tumours (*n*=45) (HG-U133A). (**B**) ISG15 gene expression in normal urothelium (*n*=18), Ta tumours (*n*=28), T1 tumours (*n*=20), and T2–T4 tumours (*n*=45) (EOS Hu03 microarray).

We performed a gene expression profiling analysis using microarrays and determined that the ISG15 transcript was associated with bladder cancer. Comparison of ISG15 gene expression in nine normal urothelium samples and 45 Ta tumours revealed a 2.7-fold increase in ISG15 transcript expression in Ta tumours (*P*=1.31E−05) (Figure 1A). To validate this finding, and to extend the study to also include samples from invasive tumours, the ISG15 gene expression profiling analysis was repeated on an independent sample set consisting of 18 normal urothelium samples, 28 Ta, 20 T1, and 45 T2–T4 tumours (Figure 1B). These samples were analysed using custom Affymetrix arrays (Eos Hu03 microarray). In agreement with the first microarray study, the ISG15 transcript was increased in Ta tumours (1.4-fold, *P*=5.00E−05), in T1 tumours (1.7-fold, *P*=4.14E-08), and to the greatest extent in T2–T4 tumours (2.0-fold, *P*=4.12E-13) compared to normal bladder. The median expression of ISG15 transcripts increased in 93% (26 out of 28) of the Ta tumours, 100% (20 out of 20) of the T1 tumours, and 98% (44 out of 45) of the T2–T4 compared to the median expression of the 18 normal tissue samples. ISG15 has been implicated in the primary immune response to infection by microbial pathogens; however, we did not find any correlation between increased expression of ISG15 and inflammation of the bladder (Student's *t*-test, *P*>0.1). The level of inflammation was determined by two independent methods: pathological examinations of random bladder biopsies, and testing for bacterial infections using the urine Multistix® 7 test (Bayer A/S Diagnostics Kgs. Lngby, Denmark), which measures the nitrite and leucocyte levels (Robertson and Duff, 1988). These findings suggested that increased ISG15 expression is associated with bladder cancer rather than an associated immune response.

### ISG15 protein expression

**Figure 2:**
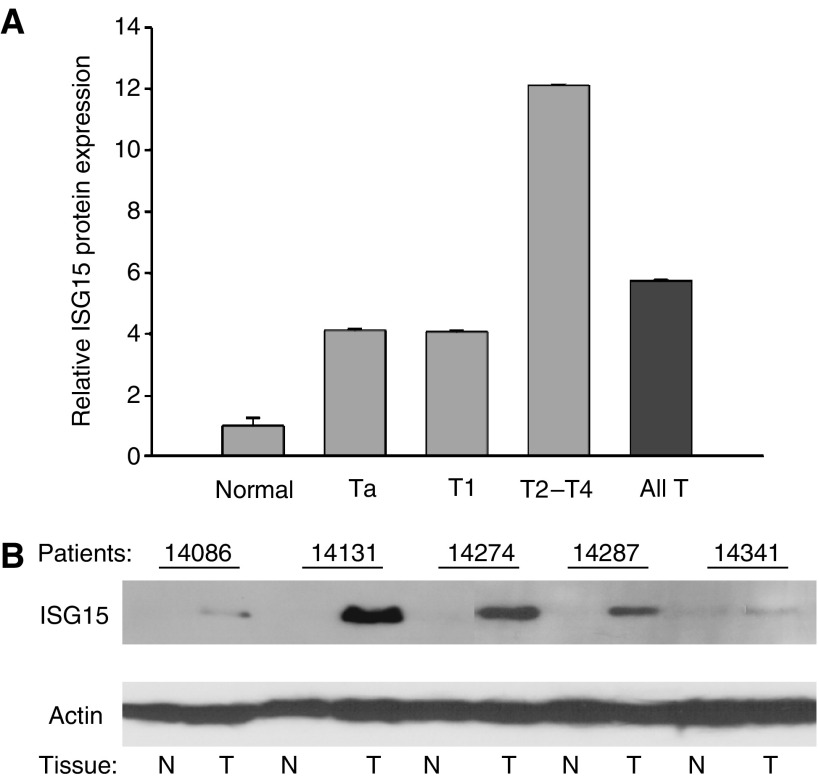
(**A**) Densitometric analysis of ISG15 protein expression by Western blotting analysis. The relative expression of each tumour group compared to the normal level is shown in the diagram (All T=all tumours). All ISG15 levels are normalised to actin expression. (**B**) Western blotting analysis of ISG15 protein expression in 10 paired samples; T2–T4 tumour (T) and normal bladder (N) from the same cancer patient (upper panel). The actin protein expression was measured as a control for equivalent protein loading (lower panel).

Microarray analysis measures the level of mRNA, however, protein expression may be a more accurate measure of cellular function, and does not always mirror mRNA expression. To determine if the bladder cancer-associated increase in ISG15 mRNA corresponded to an increase in ISG15 protein expression, tissue biopsies from 25 patients diagnosed with different stages of bladder cancer and 10 normal individuals were analysed by Western blotting. ISG15 expression was quantified by densitometric analysis, and the values were normalised to the constitutively expressed beta-actin protein. The Western blotting analysis revealed consistently increased levels of ISG15 protein in bladder cancer compared to normal samples (*P*<0.05) (Figure 2A and Table 1). Importantly, a comparison of ISG15 expression across different stages of bladder tumours revealed a significant increase in ISG15 protein level with advancing tumour stage. The ISG15 protein levels increased 4.1-fold in Ta and T1 tumours, and as much as a 12.1-fold increase in T2–T4 tumours (Table 1). Only a single 15 kDa protein band representing unconjugated ISG15 protein was observed in Western blots (see Figure 2B), and high molecular weight bands representing ISG15-modified cellular proteins were not observed (not shown). These results indicate that ISG15 protein expression is significantly increased in individuals diagnosed with bladder cancer, and suggest that ISG15 is most highly expressed in muscle invasive disease.

The analyses described in Figures 1 and 2A utilised normal tissue obtained from individuals with no history of bladder cancer, and thus may reflect the variation in basal ISG15 expression in the population at large. To compare ISG15 expression in normal bladder and bladder tumour samples that have an identical genetic background, biopsies were obtained from the tumour and from an adjacent, histologically normal, region of the same bladder from 29 patients with late stages of bladder cancer (T2–T4). Western blot analysis of these samples revealed that, similar to the comparison of ISG15 expression in normal individuals and cancer patients, unconjugated ISG15 was increased in tumour as compared to normal urothelium from the same patient. A representative Western blot analysis of five patients is shown in Figure 2B. These findings indicate that ISG15 protein overexpression is a tumour-specific event.

### Immunohistochemical analysis of ISG15 expression

Tumour biopsies contain a heterogeneous mixture of cells including tumour, endothelial, and immune cells; therefore, gene expression in these samples represents the sum of contributions from distinct cell types. In order to characterise the ISG15 expression pattern in normal urothelium and bladder tumours, we performed a series of immunohistochemical stainings. ISG15 expression was evaluated by staining paraffin-embedded tissue sections with specific polyclonal antibody raised against ISG15 protein in rabbit. In total, ISG15 expression was analysed in both complete biopsy samples (four Ta, and five T2–T4 tumours), and in tissue microarray samples (20 Ta, and 20 T2–T4 tumours). To evaluate the ISG15 expression under normal conditions, 10 normal urothelium samples were stained with ISG15 antibody; these samples were also included in the tissue microarray. The antibody was tested for specificity for ISG15 by Western blotting analysis (Supplementary Figure 1), and it only recognised one protein band identical to ISG15 protein with the expected molecular weight of approximately 15 kDa.

**Figure 3:**
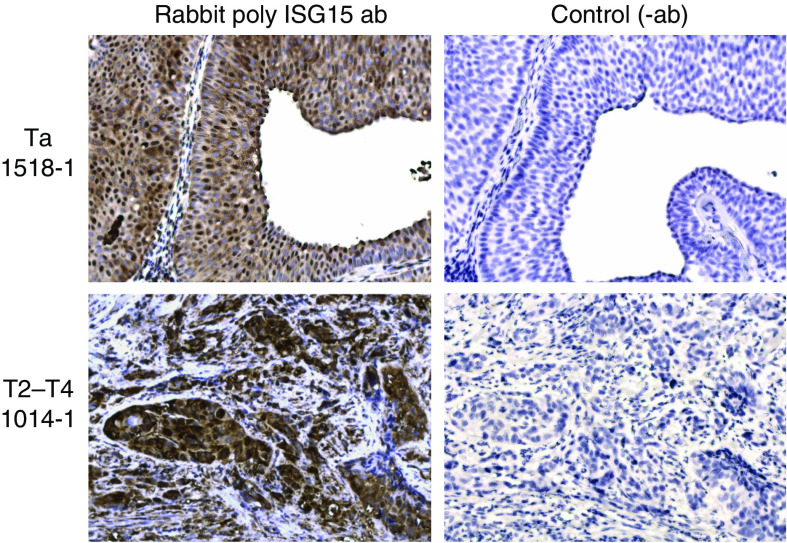
Immunohistochemical staining of ISG15 expression in bladder tumours. Tissue material obtained from two patients; 1518-1 (Ta tumour) and 1014-1 (T2–T4 tumour). Original magnification: × 20.

**Figure 4:**
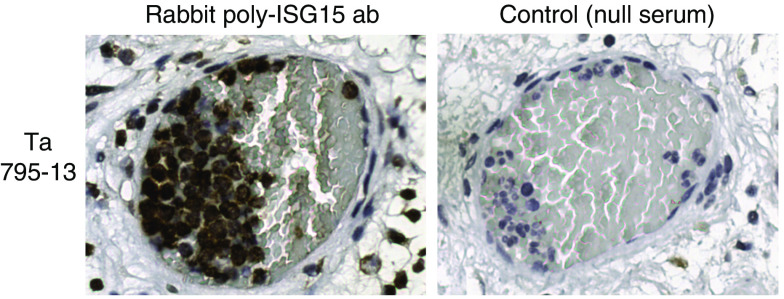
A tumour-infiltrating blood vessel with ISG15-positive immune cells. Tissue material obtained from patient 795-13 with a Ta tumour. Original magnification: × 40.

**Figure 5:**
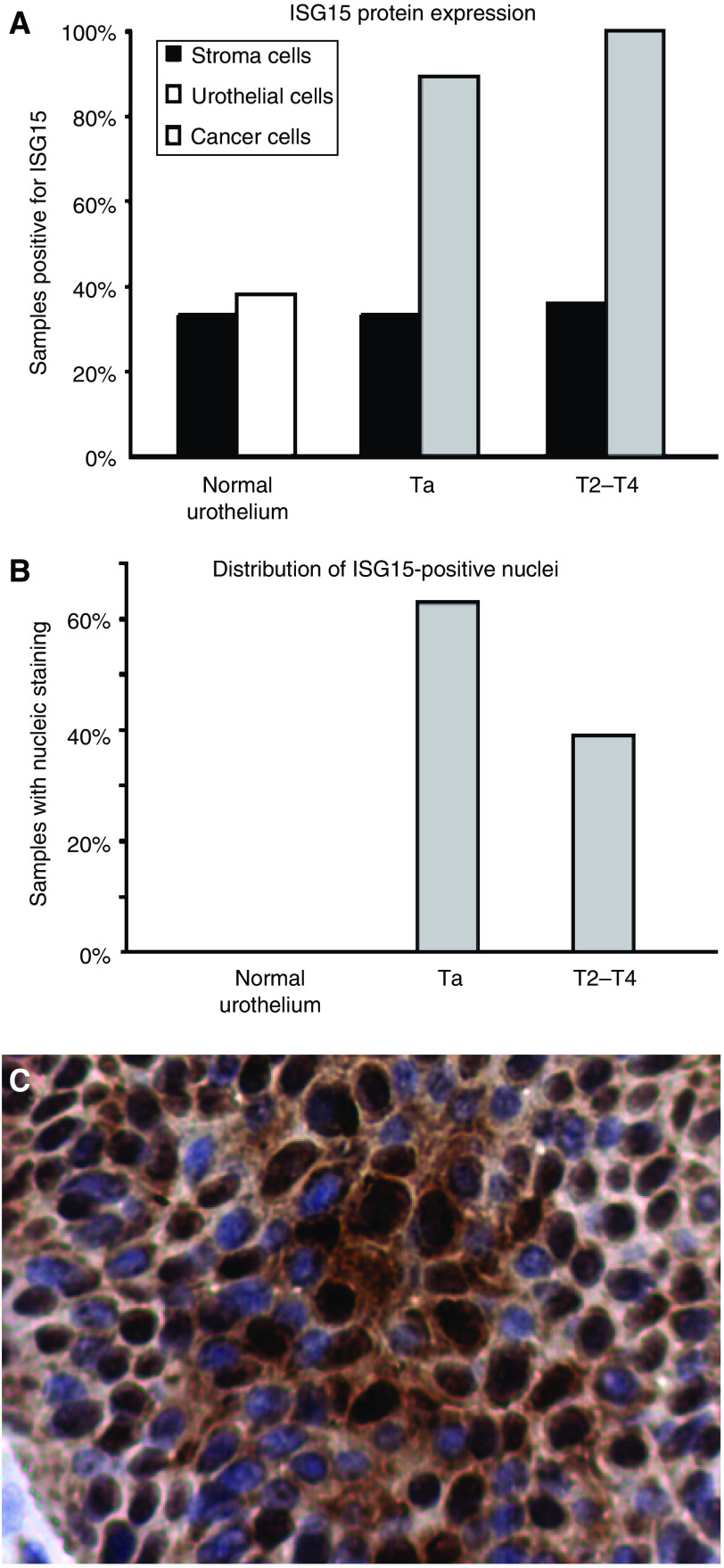
(**A**) Immunohistochemical analysis of ISG15 expression using tissue microarray normal urothelium: *N*=10; Ta tumours: *N*=20; T2–T4 tumours: *N*=20. (**B**) Distribution of samples with ISG15-positive nuclei (>50%) among normal urothelium samples, Ta tumours, and T2–T4 tumours. (**C**) Nuclear ISG15 expression of a Ta tumour. Original magnification: × 40.

Staining of bladder tumour sections with antibody specific to ISG15 revealed specific signal for ISG15 in both cancer cells and stromal immune cells (Figures 3 and 4). As negative controls, either slides without primary antibody or stained with rabbit null serum were included; both methods resulted in no staining, as expected (Figures 3 and 4). Figure 4 shows a tumour-infiltrating blood vessel that contains several strongly ISG15-positive immune cells. The exact lineage of those immune cells remains to be determined. The two tumours shown in Figure 3 mirror our previous findings that T2–T4 tumours express more ISG15 protein than Ta tumours as seen by Western blotting analysis. Compared to normal urothelium, significantly more tumours display ISG15 expression, as 100% (18 out of 18) of the T2–T4 and 89% (17 out of 19) of the Ta tumours stained positive for ISG15 protein (*χ*^2^-test, *P*<0.05). Only 38% (three out of eight) normal urothelium samples were positive (Figure 5A). A significant change in the staining pattern of stromal cells was not observed when we compared normal urothelium with tumours, indicating that ISG15 expression in this cell type is not associated with bladder cancer. Supplementary Figure 2 shows normal urothelium, Ta, and T2–T4 tumours stained with ISG15 antibody. Interestingly, our analysis revealed that not only did the fraction of samples staining positive for ISG15 increase in tumours, but the subcellular staining pattern also differed in tumour as compared to normal urothelium. A significant number of the tumours displayed nuclear staining by ISG15 (63% Ta tumours, and 39% T2–T4 tumours), whereas this phenomenon was not seen in normal urothelium (*χ*^2^-test, *P*<0.05) (Figure 5B).

### ISG15 gene correlation analysis

Inflammation of the bladder wall is frequently observed in bladder cancer patients. In this study, 64% of the patients were found positive for local inflammation by pathological examination of random bladder biopsies. Interferons are proinflammatory cytokines that induce high levels of ISG15 expression; therefore, we sought to determine if bladder cancer-associated ISG15 expression may originate from a general response to IFN-mediated inflammation. To do this, a correlation analysis was performed to search for genes that correlated with ISG15 gene expression based on the microarray data previously generated. The ISG15 gene was found to correlate strongly with a group of IFN-inducible immune-related genes. Only genes having correlation coefficients higher than 0.410 were considered significant (*P*<0.001, *N*=54, HG-U133A microarrays). For further information see Materials and Methods. A subset of the significantly correlated genes is shown in Table 2. In order to validate this finding, the correlation coefficients to the ISG15 gene in the validation data set generated by the custom affymetrix microarray (*N*=111, EOS Hu03 microarrays) were measured. All of the genes shown in Table 2 were repeatedly found to correlate with ISG15 in these independent samples. As a control, the probeset for the beta-actin gene was included, and it did not show a significant correlation to ISG15 as expected. To extend the analysis, the expression of known pro- and anti-inflammatory genes were examined; however, none of those genes showed a significant positive correlation to ISG15. These findings suggest that ISG15 dysregulation in bladder cancer does not appear to reflect a generalised inflammatory response as changes in pro- and anti-inflammatory markers between normal and tumour samples are insignificant.

## DISCUSSION

We have performed microarray analysis in combination with Western blotting and immunohistochemical staining to study ISG15 expression in a large population of bladder cancer patients. Interferon-stimulated gene 15 kDa was differentially expressed in normal urothelium and bladder tumours, as it exhibited significant upregulation in early-stage tumours, and displayed a further increase in expression, which correlated with progression to a muscle invasive T2–T4 stage. This pattern of expression was observed in analyses using two independent microarray platforms, indicating that ISG15 expression is strongly associated with bladder cancer. Previously identified bladder cancer markers have been of limited utility in clinical applications owing to the variable (*HRAS*; Fitzgerald *et al*, 1995) or low (<20%; *CCND1*; Hovey *et al* (1998); and *ERBB2*; Bringuier *et al* (1996) frequency of altered expression or activity in tumours. *CDC91L1* was recently identified as an oncogene that is overexpressed in >30% of bladder cancer cell lines and primary tumours, making it one of the most commonly altered oncogenes found in bladder cancers (Guo *et al*, 2004). In comparison, we found the ISG15 protein upregulated in nearly all of the tumours analysed in the study; it was overexpressed in 93% (26 out of 28) of Ta tumours, 100% (20 out of 20) of T1 tumours, and 98% (44 out of 45) of T2–T4 compared to the normal tissue samples.

In good agreement with the microarray results, the level of ISG15 protein was also increased in tumours. The levels of expression were highly stage-associated, as ISG15 protein increased through the tumour stages Ta, T1, to T2–T4. The mean levels in T2–T4 tumours were increased approximately 12-fold compared to normal tissue. A general increase in ISG15 protein in individual cancer cells of late-stage tumours, and the presence of considerably more ISG15-positive stromal immune cells in late stage as compared to Ta tumours was observed by immunohistochemical staining, and both of these factors likely contributed to the large increase in ISG15 expression.

Interferon-stimulated gene 15 kDa is normally expressed at low, basal levels in the absence of a specific inducer; therefore, we examined the microarray data for signalling components and effector genes that may reveal the gene induction pathway responsible for enhanced ISG15 expression in bladder tumours. Interferon expression was not detected in the bladder tumour samples analysed; however, a Pearson correlation analysis revealed that ISG15 expression was significantly correlated with a group of IFN-induced genes involved in the immune response. Moreover, the expression of several pro- and anti-inflammatory genes was unchanged between normal and bladder tumour tissue, and did not correlate with ISG15 expression. In agreement with this analysis, we did not find any correlation between ISG15 expression and inflammation in the patients in the study. Thus, despite its established role as a primary immune response gene, the ISG15 expression in bladder cancer appears to be independent of associated bladder inflammation (Loeb and Haas, 1992; Kim and Zhang, 2003). These findings suggest that ISG15 and the subset of correlating inflammation mediators may represent a specific host response to bladder cancer, rather than a generalised inflammatory response.

Staining of bladder tumour sections and paired normal and tumour samples obtained from the same bladder cancer patient with antibody specific to ISG15 protein revealed a specific expression of ISG15 protein in cancer cells. Other cells types, such as stromal immune cells, did also stain positive for ISG15. These cells may arise from the many blood vessels present in the tumour tissue; in support of this prediction, ISG15-positive cells were observed within tumour-infiltrating blood vessels. Interestingly, a significant fraction of ISG15 protein was localised to the nuclei of cancer cells, whereas ISG15 protein exhibited a diffuse cytoplasmic staining pattern in ISG15-positive stromal cells. This compartmentalisation of ISG15 may reflect tumour-associated nuclear functions of ISG15 or the presence of ISGylated cellular proteins in the nucleus. Indeed, several nuclear proteins are modified by ISG15 (Giannakopoulos *et al*, 2005; Zhao *et al*, 2005). The mechanistic basis and functional consequences of nuclear ISG15 require further study.

The tissue microarray analysis of ISG15 expression revealed that the fraction of ISG15-positive samples was increased in the groups of Ta and T2–T4 tumours compared to normal urothelium. One hundred per cent of T2–T4 tumours stained positive for ISG15, whereas only approximately 40% of the normal samples stained for ISG15. These findings demonstrate a strong correlation between enhanced ISG15 expression and bladder cancer. Further studies are required to evaluate the prognostic and diagnostic potential of ISG15 and to address the potential role of ISG15 in the malignant phenotype.

## Figures and Tables

**Table 1:** ISG15 protein expression in normal and bladder tumor samples

**Group**	**Mean±s.d.^a^**	***P*-value (*t*-test)^b^**
Normal (*n*=10)	1.0±0.25	—
Ta (*n*=15)	4.1±0.06	*P*<0.05
T1 (*n*=5)	4.1±0.05	*P*<0.05
T2–T4 (*n*=5)	12.1±0.02	*P*<0.05
All tumours (*n*=25)	5.7±0.04	*P*<0.05

ISG15=interferon-stimulated gene 15 kDa; s.d.=standard deviation; Ta=benign mucosal tumours; T1=submucosal invasive tumours; T2–T4=tumours with distal metastases.

a Normalised means of ISG15 protein expression determined by densitometry analysis of Western blotting (normal=1).

b Pairwise comparisons between the normal group and each tumour group using Student's *t*-test.

**Table 2:** ISG15 expression correlation analysis

		**Pearson's correlation coefficient (*ρ*)^§^**		
**Gene transcript**	**Gene symbol**	**HG-U133A (*N*=54)**	**EOS Hu03 <*N*=111)**	**IFN induction**
*Highly l5G15-correlating genes*				
Interferon, alpha-inducible protein (clone IFI-15K)	*GIP2*	1.00	1.00	Type 1-2
Myxovirus (influenza virus) resistance 1, interferon-inducible protein p78 (mouse)	*MX1*	0.33	0.84	Type 1-2
Hypothetical protein FLJ20035	*FLJ20035*	0.82	0.68	n.d
Proteasome (prosome, macropain) activator subunit 2 (PA23 beta)	*PSME2*	0.81	0.73	Type 2
Myxovirus (influenza virus) resistance 2 (mouse)	*MX2*	0.79	0.61	Type 1-2
Interferon regulatory factor 7	*IRF7*	0.79	0.65	Type l
Hypothetical protein FU22693	*FLJ22693*	0.79	0.71	n.d
Caspase-1, apoptosis-related cysteine protease (interleukin-1. beta, convertase)	*CASP1*	0.78	0.43	Type 2
Interferon-induced protein with tetratrico peptide repeats 1	*IFIT1*	0.76	0.56	Type l
2′-5′-Oligoadeny(late synthetase 3, 100 kDa	*OAS3*	0.76	0.75	Type1-2
Interferon-induced protein with tetratricopeptide repeats 4	*IFIT4*	0.76	0.67	Type l
Major histocompatibility complex, class 1, F	*HLA-F*	0.76	0.42	Type 2
Indoleamine-pyrrole 2,3 dioxygenase	*INDO*	0.75	0.37	Type 2
*Proinflammatory genes*				
Interleukin-1, beta	*IL1B*	0.40^#^	0.07^#^	
Interleukin-1, alpha	*IL1A*	0.01^#^	0.23^#^	
Interleukin-6 (interferon, beta 2)	*ILG*	0.13^#^	0.38	
Interferon, gamma	*IFNG*	0.09^#^	0.25^#^	
*Anti-inflammatory genes*				
Interleukin-13	*IL13*	−0.42	−0.09^#^	
Transforming growth factor, beta 1 (Camurati–Engelmann disease)	*TGFB1*	0.20^#^	−0.18^#^	
*Control gene*				
Actin, beta	*ACTS*	0.27^#^	0.24^#^	

IFN=interferon; IL=interleukin;TGF=transforming growth factor.

^§^Calculated Pearson's correlation coefficients (ρ) of significantly ISGlS-correlating genes (*P*<0.00l). The levels of significance are *P*>0.41 (HG-U133AJ, and *P*>0.29 (EOS Hu03). Genes that do not significantly correlate with ISG1S are marked by #. The ACTS gene is shown as a control.
