# Supplementary material for: Stage-associated overexpression of the ubiquitin-like protein, ISG15, in bladder cancer
Source: Br J Cancer. 2006 Apr 25;94(10):1465–71. doi: 10.1038/sj.bjc.6603099 (PMC2361278; doi:10.1038/sj.bjc.6603099)
Supplement: Supplementary Figures [file 94-6603099x1.doc]

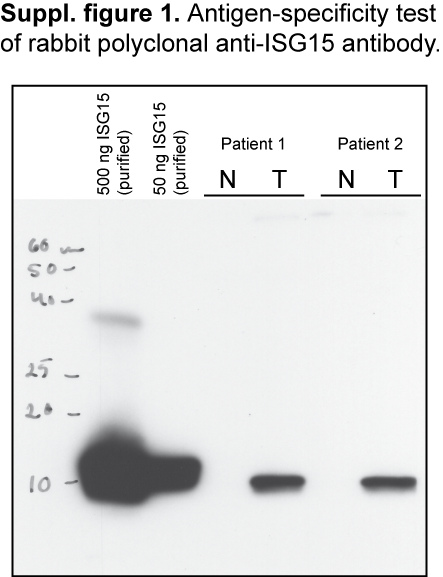


**Suppl. Figure 1.** Antibody test for ISG15 specificity by western blot analysis. The rabbit polyclonal ISG15 antibody correctly recognize both purified ISG15 protein and ISG15 protein expressed in two different tumors. No ISG15 protein was expressed in normal samples from the same patients. ISG15 protein has a molecular weight of approximately 15 kDa.


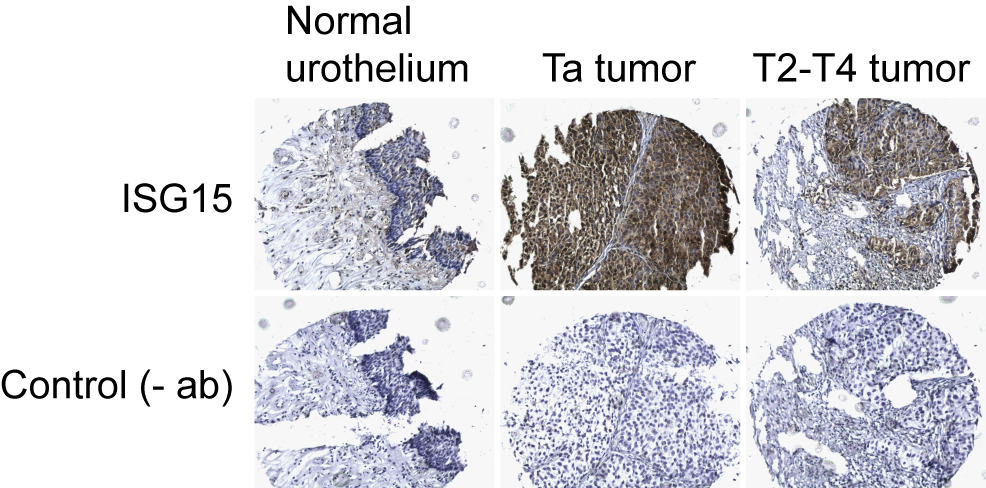


**Suppl. Figure 2.** Tissue microarray analysis of ISG15 expression of normal urothelium, Ta tumor, and T2-T4 tumor.
